# Effect of Heat-Killed *Lactiplantibacillus plantarum* SNK12 on Sleep Quality and Stress-Related Neuroendocrine and Inflammatory Biomarkers in Adults: A Randomized, Double-Blind, Placebo-Controlled, Parallel-Group Trial

**DOI:** 10.3390/life16010026

**Published:** 2025-12-24

**Authors:** Takumi Watanabe, Shiho Kurosaka, Yuriko Namatame, Toshio Kawahara

**Affiliations:** 1 College of Life and Health Sciences, Chubu University, 1200 Matsumoto, Kasugai 487-8501, Aichi, Japan; toshi@fsc.chubu.ac.jp; 2 Bio-Lab Co., Ltd., 2-1-3 Komagawa, Hidaka 350-1249, Saitama, Japan; s-kurosaka@bio-ken.jp (S.K.); yuri@bio-ken.jp (Y.N.)

**Keywords:** sleep quality, heat-killed lactic acid bacteria, postbiotics, gut–brain axis, hypothalamic–pituitary–adrenal (HPA) axis, tumor necrosis factor-α, cortisol, randomized controlled trial

## Abstract

Heat-killed *Lactiplantibacillus plantarum* SNK12 (SNK), isolated from a traditional Japanese fermented food, has been suggested to influence sleep quality, but human data on sleep improvement with heat-killed lactic acid bacteria (postbiotics) remain limited. We conducted a randomized controlled trial to test whether heat-killed SNK (≥1 × 10^11^ cells/day for 4 weeks) improves sleep quality and alters stress-related immune and neuroendocrine biomarkers. Healthy adults received SNK or a placebo for 4 weeks. The primary outcome was the Oguri–Shirakawa–Azumi Sleep Inventory MA version (OSA-MA) factor “Sleepiness on Rising”; secondary outcomes were other OSA-MA factors and the stress-related biomarkers salivary cortisol and plasma tumor necrosis factor-α (TNF-α). Compared with placebo, SNK improved Sleepiness on Rising (*p* = 0.032) and Initiation and Maintenance of Sleep (*p* = 0.010). Salivary cortisol (*p* = 0.016) and plasma TNF-α (*p* = 0.037) were also lower with SNK, and no safety concerns emerged. These concomitant changes in subjective sleep indices and stress-related biomarkers are consistent with modulation of hypothalamic–pituitary–adrenal axis activity and inflammatory pathways along the gut–brain axis. SNK may, therefore, represent a practical postbiotic option to support sleep quality.

## 1. Introduction

Sleep is essential for maintaining physical and mental health. Insomnia and poor sleep quality have been linked to an increased risk of lifestyle-related diseases, cognitive disorders, and compromised immune function [1,2]. Although pharmacotherapy is widely used, concerns regarding its side effects and dependence have driven interest in safe, food-based approaches.

Lactic acid bacteria (LAB) have long been incorporated into the human diet through fermented foods. In addition to their effects on the intestinal environment, LAB exhibit diverse physiological activities, including immune modulation, protection against infection, and influence on metabolism and neurological function [3,4]. Heat-killed LAB, often categorized as postbiotics, biogenics, or paraprobiotics, exhibit a broad range of biological activities, and their favorable safety and stability profiles have attracted attention as functional food ingredients [5]. In addition to these applications, heat-killed LAB also provides useful tools for investigating how microbial components influence neuroendocrine and immune pathways relevant to stress and sleep regulation.

*Lactiplantibacillus plantarum* SNK12 (SNK) is a bacterial strain isolated from traditional Japanese fermented foods. SNK has shown functionality in basic and clinical studies as a heat-killed preparation. Animal studies have reported antiviral effects in influenza infection models [6], enhancement of small-intestinal digestive enzyme activity [7], and increased expression of neurotrophic factors and GABA receptors, accompanied by improved behavioral responses in mice subjected to social defeat stress [8]. These findings from animal studies are mechanistic and are best regarded as hypothesis-generating and helpful for guiding the design of human clinical trials. A randomized, placebo-controlled, double-blind trial demonstrated the attenuation of transient stress by SNK in healthy adults [9]. These findings suggest that SNK influences psychophysiological functions via the immune, digestive, and nervous systems, including stress-related neuroendocrine regulation, and could therefore affect sleep quality.

Several food components have been investigated for their sleep benefits, including γ-aminobutyric acid (GABA), which may facilitate sleep onset and improve sleep architecture [10]; L-theanine, which exerts relaxation effects [11]; and fermented milk or probiotics, which have been linked to improvements in sleep-related indices [12,13]. However, evidence for the sleep benefits of heat-killed LAB remains limited; most reports have focused on *Lactobacillus brevis* SBC8803 in animals and small human studies [14,15]. Given that SNK modulates stress responses and neurochemical factors, it is a plausible candidate for improving sleep. At a mechanistic level, any potential sleep-related effects of non-viable lactobacilli are more likely to be mediated by microbe-associated structural components—such as peptidoglycan, lipoteichoic acid, and exopolysaccharides—that engage host pattern-recognition receptors and downstream immune–neuroendocrine signaling, rather than by bacterial replication [16,17]. Human evidence is still heterogeneous; nevertheless, randomized trials and a recent meta-analysis have associated non-viable lactobacilli with improvements in subjective sleep under psychological stress [12]. Against this background, we tested whether SNK improves sleep quality and stress-related biomarkers in healthy adults with mild complaints.

Accordingly, we tested heat-killed SNK in a randomized controlled trial, combining OSA-MA with stress-related biomarker assessments. Therefore, in this trial, we aimed to determine whether SNK intake improves sleep quality in adults. Specifically, the primary outcome was the OSA-MA factor Sleepiness on Rising [18]. The secondary outcomes were the OSA-MA factors: Initiation and Maintenance of Sleep, Frequent Dreaming, Refreshing Sleep, and Sleep Length. Additionally, to assess physiological responses relevant to sleep insufficiency and chronic stress, we measured salivary cortisol as an index of hypothalamic–pituitary–adrenal (HPA) axis activation, plasma tumor necrosis factor-α (TNF-α), and interleukin (IL)-6 as inflammatory markers [19,20,21].

## 2. Materials and Methods

### 2.1. Ethics

Healthy Japanese adults were enrolled at Takara Clinic (Seishinkai Medical Corporation, Tokyo, Japan). The protocol complied with the Declaration of Helsinki (2013 revision) and the Ethical Guidelines for Medical and Life Science Research Involving Human Participants and was approved by the Ethics Review Committee of Takara Clinic, Seishinkai Medical Corporation (Approval No.: 2407-01660-0126-16-TC) on 10 July 2024. The trial was registered in the UMIN Clinical Trials Registry (UMIN-CTR; UMIN000055110) on 30 July 2024. Written informed consent was obtained from all the participants.

### 2.2. Participants

Eligibility targeted adults who reported morning fatigue or dissatisfaction with sleep quality in daily life and who showed a low score on the “Sleepiness on Rising” factor of the OSA Sleep Inventory MA version (OSA-MA) [18]. Only individuals without clinically relevant issues on the Beck Depression Inventory–II (BDI-II) were included [22]. The Beck Depression Inventory–II is a standardized self-administered questionnaire that yields a total score reflecting depressive symptom severity. It was used at screening to exclude individuals with clinically relevant depressive symptoms, to reduce potential confounding of subjective sleep outcomes and stress-related biomarkers and to ensure participant safety. The key exclusion criteria were malignant tumors, serious chronic diseases (e.g., heart disease and cerebrovascular disorders), immunodeficiency, ongoing treatment for sleep disorders, current intake of Foods for Specified Health Uses (FOSHU) or Foods with Function Claims (FFC), pregnancy or lactation, and irregular sleep patterns due to shift or night work.

### 2.3. Test Supplements

The test supplement was a granulated preparation containing heat-killed *Lactiplantibacillus plantarum* SNK12. Heat killing was performed by heating a bacterial suspension at 105 °C for 30 min using an autoclave, as described previously [6]. Each packet contained 50 mg of heat-killed bacterial powder (≥1 × 10^11^ cells) and 950 mg of dextrin. The placebo matched the formulation without bacterial powder (dextrin 1000 mg) and was indistinguishable in terms of appearance, shape, color, and flavor. Participants consumed one packet daily with water or lukewarm water for four consecutive weeks. This dose level (≥1 × 10^11^ cells/day) was prespecified based on prior human experience with SNK, indicating good tolerability at this level [9] and on alignment with the range commonly used in adult trials of heat-killed lactobacilli (approximately 10^10^–10^11^ cells/day) [14,15].

Compliance was assessed using packet counts and participant diaries. Cell counts per packet were verified by DAPI staining (Dojindo, Kumamoto, Japan) and observation under a fluorescence microscope (Olympus, Tokyo, Japan).

### 2.4. Study Design

This was a randomized, double-blind, placebo-controlled, parallel-group trial. Participants were randomized 1:1 to the test or placebo groups using a variable-block randomization list with random block sizes of 4–8, generated using R (version 4.4.1; blockrand package version 1.5; the R Foundation for Statistical Computing, Vienna, Austria). Allocation codes were strictly concealed by an independent allocation manager, who was not otherwise involved in trial conduct, outcome assessment, or data analysis, and investigators, participants, outcome assessors, testing laboratories, and data analysts were blinded. The code was opened after trial completion and database lock.

The intervention lasted 4 weeks and included three visits: screening, baseline, and week 4. Administration of OSA-MA and biospecimen collection were scheduled at similar times of day across visits, and the collection time was recorded at each visit. Participants were instructed to standardize pre-visit lifestyle factors (alcohol, caffeine, exercise, and bedtime) on the preceding day. These factors were confirmed by participant self-report through a brief interview at each visit, and any deviations were recorded when reported; adherence was not objectively or quantitatively measured.

Manufacturers printed group-identification marks on each outer pouch, and only the allocation manager retained the allocation list until the database lock. This study followed our previously published randomized, double-blind, placebo-controlled SNK trial [9].

### 2.5. Measurements

Venous blood was drawn from the median cubital vein and centrifuged to obtain plasma. Saliva was collected in a seated position using Salivette devices (Sarstedt, Nümbrecht, Germany) at matched time windows at screening and week 4. Samples were promptly cooled, stored at −80 °C, and batch-analyzed.

Plasma TNF-α was measured using the Quantikine High Sensitivity ELISA (R&D Systems, Minneapolis, MN, USA), IL-6 using the Human IL-6 ELISA (FUJIFILM Wako Pure Chemical Corp., Osaka, Japan), and salivary cortisol using the Salivary Cortisol EIA (Yanaihara Institute Inc., Shizuoka, Japan). Salivary cortisol is widely used as a noninvasive indicator of stress responses and circadian rhythms [23,24,25] and has been associated with work-related stress and sleep quality [26]. All assays were performed in duplicate; standard-curve fit and control acceptability were verified.

### 2.6. Primary Outcome

The primary outcome was the OSA-MA “Sleepiness on Rising” factor score at week 4. The OSA-MA, specifically developed and standardized for Japanese adults, comprises five factors—Sleepiness on Rising, Initiation and Maintenance of Sleep, Frequent Dreaming, Refreshing Sleep, and Sleep Length—each expressed as a response-scale value (Zc score) [18].

### 2.7. Secondary Outcomes

Secondary outcomes were the remaining OSA-MA factor scores (Initiation and Maintenance of Sleep, Frequent Dreaming, Refreshing Sleep, and Sleep Length), total OSA-MA score across all 16 items, plasma inflammatory cytokines (TNF-α and IL-6), and salivary cortisol concentration.

### 2.8. Safety Endpoints

Safety analyses were conducted in the Safety Analysis Set (SAF), defined as all randomized participants who ingested at least one dose and provided any safety data. Adverse events (AEs) were collected from the first dose through the week-4 visit via structured interviews at each visit. The primary safety endpoint was the incidence of treatment-emergent AEs (TEAEs), summarized as the number and percentage of participants with ≥1 AE in each group. AE severity (mild, moderate, or severe) and causality to the investigational product (unrelated, unlikely, possibly, probably, definitely related, or unknown) were assessed by investigators, and terms were grouped by organ-system categories.

The secondary safety endpoints comprised changes from baseline to week 4 in (i) urinalysis (protein, glucose, pH, occult blood), (ii) peripheral blood tests (complete blood count and serum biochemistry), (iii) blood pressure (systolic/diastolic), and (iv) anthropometrics (height, weight, body mass index [BMI]). Clinically meaningful abnormalities and shifts from baseline were reviewed descriptively.

Between-group comparisons of AE incidence used the χ^2^ test or Fisher’s exact test, as appropriate, and risk differences with 95% confidence intervals were calculated. All statistical tests were two-sided with α = 0.05 (*p* < 0.05 considered significant). No imputation was performed on the safety data.

### 2.9. Statistical Analysis

The sample size was set at 50 participants (25 per group) as the maximum feasible within the study budget. To allow for attrition, three additional participants per group were enrolled, yielding a total of 56 participants (28 per group).

Analyses were performed using IBM SPSS Statistics for Windows, version 23 (IBM Japan, Ltd., Tokyo, Japan) [27]. Baseline characteristics (age, height, weight, BMI, systolic/diastolic blood pressure, pulse rate, and BDI-II total score) were compared between groups using Welch’s t-test for continuous variables and the χ^2^ test for sex [28]. After trial completion, the principal investigator reviewed all cases for adherence to the inclusion/exclusion criteria and informed consent; protocol deviations and discontinuations were adjudicated in a committee meeting. Based on these reviews, the principal investigator and statistician finalized the dataset, and unblinding was performed after database lock.

The analysis populations were defined according to ICH E9 [29]; the primary analysis was conducted using the Per Protocol Set (PPS). The PPS comprised participants who met the key eligibility criteria, received the allocated intervention, had data for the primary endpoint, and had no major protocol deviations. The SAF included all participants who consumed at least one dose of the test product or placebo.

Between-group comparisons of the primary outcome (OSA-MA Sleepiness on Rising) employed analysis of covariance (ANCOVA), with group as a fixed effect and baseline as a covariate [30]. For ANCOVA models, assumptions were evaluated using residual diagnostics based on residual plots, including assessment of the residual distribution and variance homogeneity. The same ANCOVA model was applied to other OSA-MA factors (Initiation and Maintenance of Sleep, Frequent Dreaming, Refreshing Sleep, and Sleep Length). Individual OSA-MA items (16 in total) were treated as ordinal variables and compared between groups using the Mann–Whitney U test [31]. Plasma TNF-α, IL-6, and salivary cortisol were log-transformed as needed prior to ANCOVA. Safety analyses were performed using SAF. Post-intervention abnormalities in urinalysis and peripheral blood tests were compared between groups using the χ^2^ test [28]. AE incidence was summarized by group, and tests of proportions were used where appropriate. All statistical tests were two-sided with a significance level of 5%.

## 3. Results

Overall, compared with placebo, SNK intake was associated with greater improvements in the OSA-MA factors Sleepiness on Rising and Initiation and Maintenance of Sleep, alongside lower salivary cortisol and plasma TNF-α, with no safety concerns identified.

### 3.1. Participant Flow and Baseline Characteristics

**Figure 1:**
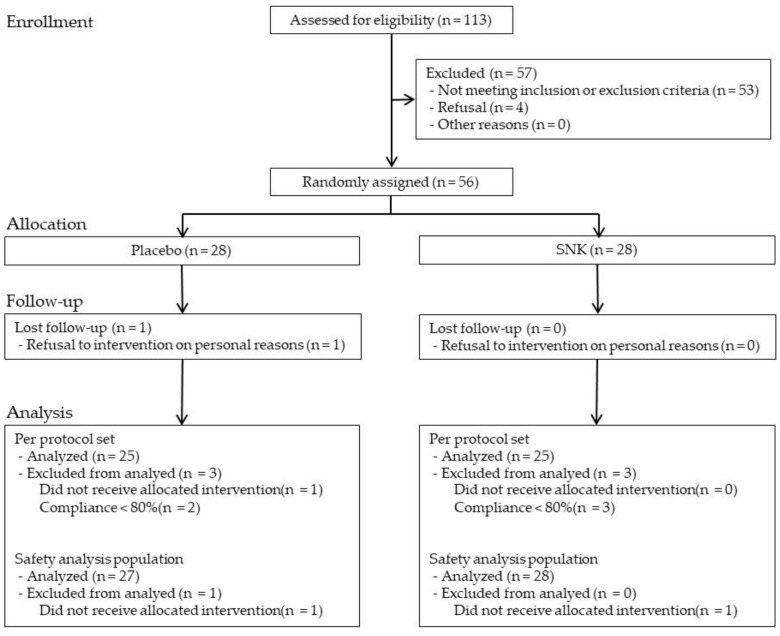
Participant flow and analysis populations. The diagram summarizes screening, randomization, exclusions, and the numbers included in the FAS, PPS, and SAF. Abbreviations: FAS, Full Analysis Set; PPS, Per Protocol Set; SAF, Safety Analysis Set.

Figure 1 outlines the participant flow and analysis sets. Of the 113 individuals screened, 56 met the eligibility criteria and were randomized in a 1:1 ratio to either the placebo group (*n* = 28) or the SNK group (*n* = 28). One participant in the placebo group did not attend the week-4 visit, and three in the SNK group and two in the placebo group had a supplement adherence rate <80%; these six participants were excluded from the Per Protocol Set (PPS). Accordingly, the PPS consisted of 50 participants (placebo, *n* = 25; SNK, *n* = 25) and served as the primary efficacy analysis set. For the Safety Analysis Set (SAF), one participant in the placebo group was excluded because no intervention was received after allocation. Thus, the SAF included 55 participants (placebo, *n* = 27; SNK, *n* = 28) and served as the Safety Analysis Set. The baseline characteristics of the PPS are summarized in Table 1, and there were no significant between-group differences in any variable. Collection time at week 4 did not differ between groups (placebo 10:34, SD 73.1; SNK 10:18, SD 39.7; *p* = 0.355).

### 3.2. OSA Sleep Inventory (OSA-MA)

In ANCOVA models adjusted for baseline and using the PPS, among the five OSA-MA factors, the primary outcome, Sleepiness on Rising, showed a significantly greater improvement at week 4 in the SNK group than in the placebo group (EMM ± SE: placebo 16.4 ± 0.8, SNK 19.1 ± 0.8; Δ = 2.6; 95% CI, 0.2 to 5.1; *p* = 0.032). The factor “Initiation and Maintenance of Sleep” also showed a significantly greater improvement in the SNK group than in the placebo group (placebo 15.4 ± 0.9, SNK 18.9 ± 0.9; Δ = 3.4; 95% CI, 0.9 to 6.0; *p* = 0.010). There were no significant between-group differences noted for Frequent Dreaming, Refreshing Sleep, or Sleep Length (Table 2).

In exploratory item-level analyses, the SNK group showed significantly greater improvements than the placebo group for Item 2 “Focused–Not focused” (*p* = 0.011), Item 7 “Often felt drowsy before falling asleep–Rarely felt drowsy” (*p* = 0.046), Item 10 “Fell asleep easily–Had difficulty falling asleep” (*p* = 0.008), and Item 16 “My sleep was light–My sleep was deep” (*p* = 0.023). Full item-level results are presented in Table S1.

### 3.3. Blood and Salivary Biomarkers

**Figure 2:**
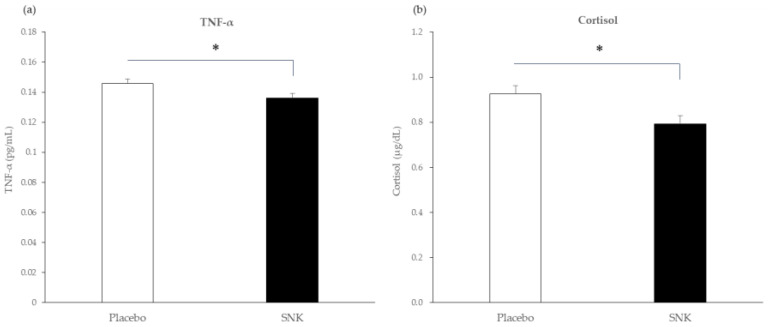
Stress-related inflammatory and neuroendocrine biomarkers at week 4. (**a**) Plasma TNF-α (pg/mL). Bars show adjusted means (EMM ± SE) from ANCOVA controlling for baseline; the between-group difference was Δ = −0.009 pg/mL (95% CI, −0.018 to −0.001), *p* = 0.037. (**b**) Salivary cortisol (µg/dL). Bars show adjusted means (EMM ± SE) from ANCOVA controlling for baseline; the between-group difference was Δ = −0.132 µg/dL (95% CI, −0.239 to −0.026); *p* = 0.016. All *p*-values are two-sided with α = 0.05. Analysis-set sizes: Placebo, *n* = 25; SNK, *n* = 25. Abbreviation: SNK, heat-killed *Lactiplantibacillus plantarum* SNK12. * *p* < 0.05.

For stress-related inflammatory and neuroendocrine biomarkers, at week 4, plasma TNF-α (EMM ± SE) was significantly lower in the SNK group than in the placebo group (placebo, 0.146 ± 0.003 pg/mL; SNK, 0.136 ± 0.003 pg/mL; between-group difference, −0.009 pg/mL; 95% CI, −0.018 to −0.001; *p* = 0.037; Figure 2a). Plasma IL-6 showed no significant between-group difference (Table S3). The stress-related neuroendocrine biomarker salivary cortisol concentration at week 4 (EMM ± SE) was significantly lower with SNK than with placebo (placebo, 0.925 ± 0.037 µg/dL; SNK, 0.792 ± 0.036 µg/dL; between-group difference, −0.132 µg/dL; 95% CI, −0.239 to −0.026; *p* = 0.016; Figure 2b).

### 3.4. Safety

In the SAF (placebo, *n* = 27; SNK, *n* = 28), no treatment-emergent adverse events were observed. No clinically relevant changes were detected in anthropometrics, physical examination, urinalysis, or peripheral blood test results (Table S2).

## 4. Discussion

In this randomized controlled trial in healthy adults, we evaluated whether ingestion of heat-killed SNK at a dose of ≥1 × 10^11^ cells/day for 4 weeks improves subjective sleep indices (Oguri–Shirakawa–Azumi Sleep Inventory MA version [OSA-MA]) and stress-related neuroendocrine and inflammatory biomarkers (salivary cortisol, plasma TNF-α). The primary endpoint was the OSA-MA factor Sleepiness on Rising; secondary endpoints were the other OSA-MA factor scores, item-level scores, salivary cortisol, and plasma TNF-α levels.

The primary findings were that the SNK group showed greater improvements than the placebo group in Sleepiness on Rising and Initiation and Maintenance of Sleep, and that salivary cortisol and plasma TNF-α were lower with SNK than with placebo. Although statistically significant, the absolute differences in salivary cortisol and plasma TNF-α were small. Therefore, these biomarker findings should be interpreted cautiously as supportive secondary findings, and their biological and clinical significance requires confirmation in larger studies with repeated measurements. No clinically relevant adverse events were observed during the intervention period.

In this trial, between-group differences were observed for OSA-MA factors as subjective sleep indicators; the stress-related biomarkers cortisol and TNF-α showed lower levels in the SNK group, and these changes were directionally consistent. A randomized, double-blind, placebo-controlled design with predefined primary and secondary endpoints reduces the influence of expectancy and observer bias and enhances the interpretability. As higher OSA-MA scores indicate better status, the directional agreement between the subjective change (increased factor scores) and the physiological change (lower cortisol and TNF-α) is consistent with convergent validity. From a psychoneuroimmunology perspective, the parallel changes in subjective sleep and stress-related neuroendocrine and inflammatory biomarkers support the view that the modulation of immune–neuroendocrine pathways is involved in the observed effects. In addition, TNF-α is a representative cytokine involved in sleep regulation. Basic and clinical research has linked its elevation to sleep disturbance and excessive sleepiness, whereas its suppression has been associated with reduced sleepiness [32,33,34,35]. In contrast, circadian dynamics and cortisol arousal responses are associated with sleep quality, and disruption of the diurnal pattern has been reported in sleep disturbance [36,37]. No between-group differences in plasma IL-6 levels were observed. Due to the trial design, blood sampling was performed in the morning. IL-6 levels show diurnal variation, being higher from night to early morning and lower in the morning [38,39]. Given this time-of-day dependence and pre-analytical factors, plasma IL-6 values are more likely to be low or below the detection limit, potentially reducing the power to detect between-group differences [38,40]. Future studies should incorporate afternoon or serial sampling to confirm the consistency of these findings. The coherent improvements in subjective outcomes, together with lower cortisol and TNF-α levels, suggest modulation of immune–neuroendocrine signaling along the gut–brain axis.

At the species level, *Lactiplantibacillus plantarum* modulates immune responses in the intestinal mucosa and, via interactions with the autonomic nervous system and HPA-axis, may influence stress-related physiology and sleep [41]. The stimulation of intestinal epithelial cells, dendritic cells, and macrophages by cell-wall components and surface polysaccharides induces anti-inflammatory cytokines and helps maintain the homeostasis of the peripheral inflammatory milieu [5,42]. These mucosal immune signals are thought to be transmitted to the central nervous system via afferent vagal pathways, in addition to humoral routes, in a stepwise mechanism that suppresses hyperactivity of stress-related circuits and stabilizes HPA-axis activity [43]. For SNK, animal studies have reported increased expression of hippocampal neurotrophic factor–related genes and receptors involved in inhibitory neurotransmission in mice [8], and in a randomized double-blind trial in adults, reduced cortisol responses during transient stress loading and alleviation of negative mood were demonstrated [9]. Furthermore, SNK has been suggested to induce immune responses following oral administration [6]. Taken together, these findings support a working model wherein SNK intake elicits gut immune responses that reduce HPA-axis hyperactivity through anti-inflammatory changes and/or altered neurotrophic signaling; downstream, cortisol and TNF-α are maintained at low levels, contributing to improvements in sleep initiation/maintenance and reduced morning sleepiness. In the present trial conducted under everyday conditions in healthy adults, improvements in subjective sleep quality were observed alongside the maintenance of low levels of these stress-related physiological indicators, in line with this model.

Reviewing the accumulated human intervention studies on lactic acid bacteria, improvements in sleep quality have been reported in multiple randomized controlled trials for live cultures, including *Lactobacillus casei* strain Shirota [12], *L. plantarum* P8 [41], and *L. paracasei* 207-27 [44]. In contrast, human evidence for heat-killed preparations is relatively scarce. A meta-analysis supported improved sleep quality with *Lactobacillus gasseri* CP2305 [45], while a pilot study suggested improvements in sleep-related outcomes with *Lactobacillus brevis* SBC8803 [15].

Considering these reports, the improvements in subjective sleep and lower levels of stress-related biomarkers observed with heat-killed SNK in this study are valuable findings that support the possibility of efficacy independent of bacterial viability. In a field where efficacy reports using live bacteria have preceded them, the presentation of consistent findings with heat-killed bacteria is clinically significant because it may expand implementation options. Moreover, from a life-science standpoint, heat-killed preparations such as SNK provide tractable models for dissecting how defined microbial components influence immune–neuroendocrine networks relevant to sleep regulation in humans.

However, the generalizability of these findings should be interpreted in the context of the study population. This trial enrolled generally healthy adults with mild sleep-related complaints; as such, the findings should not be directly generalized to patients with diagnosed insomnia, anxiety disorders, or other clinical sleep disorders. In future studies, we aim to evaluate whether SNK improves sleep in individuals with clinically relevant sleep difficulties and whether such improvements translate into better quality of life.

This trial has several limitations. Sleep outcomes were assessed using a subjective questionnaire, and objective sleep measures were not collected. The intervention period was relatively short, and the study was conducted at a single center. In addition, biomarker assessments were based on a limited sampling schedule; thus, the physiological findings should be interpreted cautiously. These limitations should be considered when interpreting the results and in the design of future studies.

Future research should include concurrent measurements of sleep stages and arousal indices using polysomnography, sleep efficiency and sleep latency using actigraphy, as well as heart rate and heart-rate variability, to accurately verify the consistency between subjective and physiological indicators. Additionally, a gut microbiota analysis will aid in comprehensively evaluating the associations between changes in the microbiota, sleep-related outcomes, inflammatory cytokines, and endocrine markers. This will clarify the sequential pathway leading from immune regulation in the gut through autonomic and HPA-axis responses to sleep improvement, including its temporal dynamics, thereby contributing to a refined understanding of the mechanisms by which lactic acid bacteria affect sleep.

## 5. Conclusions

In this trial in healthy adults, ingesting heat-killed *Lactiplantibacillus plantarum* SNK12 (SNK; ≥1 × 10^11^ cells/day for 4 weeks) produced significant improvements versus placebo in the OSA-MA factors Sleepiness on Rising and Initiation and Maintenance of Sleep. These changes were observed alongside lower salivary cortisol and plasma TNF-α levels, with no safety concerns emerging.

Taken together, these findings indicate that heat-killed SNK exerts measurable effects on subjective sleep quality in parallel with stress-related neuroendocrine and inflammatory biomarkers in healthy adult participants. From an applied perspective, SNK may act as a postbiotic that can be incorporated into daily life to support sleep quality and morning functioning. The pattern of changes is consistent with hypothesized gut–brain pathways, whereby bacterial components modulate gut immune activity and attenuate HPA-axis hyperactivity, with possible parallel influences through vagal signaling and tryptophan metabolism (serotonin/GABA related). Future studies should test these causal links by integrating time-standardized endocrine and cytokine measurements with gut microbiota and metabolite profiling (e.g., short-chain fatty acids and tryptophan metabolites), together with objective sleep indicators, such as polysomnography, actigraphy, and heart rate/heart-rate variability.

## Figures and Tables

**Table 1:** Baseline characteristics of participants.

	Placebo (*n* = 25)	SNK (*n* = 25)	*p*-Value
Sex, *n* (male/female)	10/15	9/16	1.000
Age, years	49.6 ± 9.8	48.6 ± 9.6	0.923
Height, cm	162.7 ± 9.2	161.8 ± 7.7	0.923
Weight, kg	56.5 ± 11.8	53.6 ± 11.6	0.494
Body mass index, kg/m^2^	21.2 ± 3.2	20.3 ± 3.2	0.503
Systolic blood pressure, mmHg	117.4 ± 19.7	115.2 ± 18.6	0.971
Diastolic blood pressure, mmHg	73.1 ± 11.0	74.9 ± 12.5	0.347
BDI-II total score	9.4 ± 5.3	10.2 ± 5.6	0.787
Sleepiness on rising score	14.6 ± 2.8	14.0 ± 3.1	0.786
Collection time at baseline	10:24 (SD 64.2)	10:18 (SD 41.5)	0.697

Values are presented as mean ± SD unless otherwise indicated. Comparisons between the two groups were performed using the χ^2^ test for the number of subjects (male/female). Other data are expressed as mean ± SD and were compared between groups using Welch’s t-test. Collection time is presented as mean clock time; SD is expressed in minutes. All tests were two-sided, with α = 0.05. Abbreviations: SNK, heat-killed *Lactiplantibacillus plantarum* SNK12; BDI-II, Beck Depression Inventory–II.

**Table 2:** Effects of SNK supplementation on the five factors of the OSA Sleep Inventory.

	Baseline (Mean ± SD)		Week 4 (Mean ± SD)		Adjusted Week 4 (EMM ± SE)		Between-Group Difference				
Factor	Placebo	SNK	Placebo	SNK	Placebo	SNK	Δ (SNK − Placebo)	SE	95% CI−	95% CI+	*p*-Value
Sleepiness on Rising	14.6 ± 2.8	14.0 ± 3.1	16.6 ± 5.5	18.9 ± 3.0	16.4 ± 0.8	19.1 ± 0.8	2.6	1.2	0.2	5.1	0.032
Initiation and Maintenance of Sleep	14.9 ± 3.1	14.4 ± 3.7	15.5 ± 4.7	18.8 ± 4.6	15.4 ± 0.9	18.9 ± 0.9	3.4	1.3	0.9	6.0	0.010
Frequent Dreaming	21.7 ± 6.2	21.4 ± 5.4	20.9 ± 7.1	22.1 ± 7.1	20.8 ± 1.4	22.1 ± 1.4	1.3	1.9	−2.6	5.2	0.502
Refreshing	13.7 ± 3.7	13.8 ± 3.2	17.3 ± 4.8	17.8 ± 4.5	17.4 ± 0.9	17.8 ± 0.9	0.5	1.3	−2.1	3.0	0.710
Sleep Length	15.1 ± 4.7	15.9 ± 3.3	19.3 ± 3.4	18.8 ± 4.4	19.4 ± 0.7	18.6 ± 0.7	−0.8	1.0	−2.8	1.2	0.428

Baseline and week-4 values are expressed as mean ± SD. Adjusted week-4 values are estimated marginal means (EMM ± SE) from ANCOVA with baseline as a covariate and group (Placebo, SNK) as a fixed factor. Δ indicates the between-group difference (SNK − Placebo) in adjusted means with its 95% confidence interval (CI); SE for Δ is also reported. All *p*-values are two-sided with α = 0.05. Analyses were performed using the PPS. Analysis-set sizes: Placebo, *n* = 25; SNK, *n* = 25. Abbreviations: SNK, heat-killed *Lactiplantibacillus plantarum* SNK12; OSA, Oguri–Shirakawa–Azumi Sleep Inventory.

## Data Availability

The data are not publicly available due to ethical and privacy restrictions. De-identified individual participant data (IPD), the study protocol, and a data dictionary may be made available from the corresponding author upon reasonable request and subject to approval by the participating institutions and the ethics committee (Approval No.: 2407-01660-0126-16-TC). Summary data are provided within the article and its Supplementary Materials. The trial was registered in the UMIN Clinical Trials Registry (UMIN000055110).

## Supplementary Materials

The following supporting information can be downloaded at https://www.mdpi.com/article/10.3390/life16010026/s1. Table S1: OSA-MA item scores at baseline and week 4, and adjusted week-4 means; Table S2: Safety outcomes at week 4 (urinalysis and blood chemistry); Table S3: Plasma IL-6 concentration at baseline and week 4, and adjusted week-4 means.

## Abbreviations

The following abbreviations are used in this manuscript:

AE	adverse event
ANCOVA	analysis of covariance
BDI-II	Beck Depression Inventory–II
BMI	body mass index
CI	confidence interval
EMM	estimated marginal mean
FAS	Full Analysis Set
FFC	Foods with Function Claims
FOSHU	Foods for Specified Health Uses
GABA	γ-aminobutyric acid
HPA	hypothalamic–pituitary–adrenal
IL-6	interleukin-6
LAB	lactic acid bacteria
OSA-MA	Oguri–Shirakawa–Azumi Sleep Inventory MA version
PPS	Per Protocol Set
SAF	Safety Analysis Set
SD	standard deviation
SE	standard error
SNK	heat-killed Lactiplantibacillus plantarum SNK12
TEAE	treatment-emergent adverse event
TNF-α	tumor necrosis factor-α
