# Supplementary material for: Effect of Heat-Killed Lactiplantibacillus plantarum SNK12 on Sleep Quality and Stress-Related Neuroendocrine and Inflammatory Biomarkers in Adults: A Randomized, Double-Blind, Placebo-Controlled, Parallel-Group Trial
Source: Life (Basel). 2025 Dec 24;16(1):26. doi: 10.3390/life16010026 (PMC12843449; doi:10.3390/life16010026)
Supplement: Supplementary file 1 [file life-16-00026-s001.zip › life-4037907-supplementary.pdf]

Supplementary Materials

Table S1. OSA-MA item scores at baseline and week 4, and adjusted week-4 means.

	Baseline (mean ± SD)		Week 4 (mean ± SD)		Adjusted week 4 (estimated marginal means (EMM) ± standard error (SE))		Between-group difference				
Factor	Placebo	SNK	Placebo	SNK	Placebo	SNK	Δ (SNK − Placebo)	SE	95% CI−	95% CI+	p-value
Question 1 Feeling upon awakening in the morning (good – bad)	13.7 ± 4.7	14.2 ± 4.0	17.6 ± 4.8	18.4 ± 5.2	17.7 ± 1.0	18.31 ± 1.0	0.6	1.3	-2.1	3.3	0.643
Question 2 Ability to concentrate (concentrated – not concentrated)	16.0 ± 4.1	15.2 ± 4.3	16.9 ± 6.2	20.4 ± 3.6	16.8 ± 1.0	20.5 ± 1.0	3.7	1.4	0.9	6.5	0.011
Question 3 Clear-headedness upon awakening (clear – groggy)	12.8 ± 4.2	13.1 ± 5.2	15.0 ± 4.8	17.7 ± 5.7	15.1 ± 1.0	17.7 ± 1.0	2.6	1.5	-0.4	5.5	0.089
Question 4 Feeling active upon awakening (active – inactive)	14.2 ± 3.2	12.8 ± 3.2	16.6 ± 5.9	17.5 ± 4.3	16.6 ± 1.0	17.7 ± 1.0	1.3	1.5	-1.7	4.2	0.399
Question 5 Slept soundly (well – poorly)	12.3 ± 4.2	13.3 ± 3.4	15.9 ± 6.4	16.2 ± 4.3	16.1 ± 1.1	16.0 ± 1.1	-0.1	1.5	-3.1	3.0	0.970
Question 6 Frequency of dozing before falling asleep (frequent – rare)	15.5 ± 6.5	17.2 ± 5.6	19.6 ± 5.9	18.6 ± 6.4	20.1 ± 1.1	18.1 ± 1.1	-2.0	1.5	-5.0	1.0	0.192
Question 7 Sleep initiation (good – poor)	17.3 ± 4.4	15.5 ± 5.7	15.9 ± 5.3	18.9 ± 8.0	15.4 ± 1.3	19.2 ± 1.3	3.9	1.9	0.1	7.7	0.046
Question 8 Frequency of nocturnal awakenings (frequent – rare)	13.8 ± 3.9	13.4 ± 4.6	16.0 ± 6.1	18.2 ± 4.5	15.9 ± 1.0	18.3 ± 1.0	1.3	1.5	-1.7	4.2	0.399
Question 9 Depth of sleep (shallow – deep)	22.6 ± 6.1	21.9 ± 5.8	22.2 ± 7.3	23.0 ± 7.1	22.2 ± 1.4	23.1 ± 1.4	0.9	2.0	-3.2	5.1	0.645
Question 10 Frequency of nightmares (frequent – few)	12.9 ± 5.0	12.1 ± 3.2	13.5 ± 5.8	17.6 ± 6.2	13.3 ± 1.1	17.8 ± 1.1	4.5	1.6	1.2	7.7	0.008
Question 11 Frequency of dreaming (frequent – rare)	15.1 ± 4.0	14.0 ± 3.6	18.5 ± 5.3	19.0 ± 5.4	18.3 ± 1.1	19.2 ± 1.1	0.9	1.5	-2.1	4.0	0.531

Question 12 Mood upon awakening (good – bad)	20.7 ± 6.8	20.7 ± 5.8	19.5 ± 7.5	21.0 ± 8.5	19.5 ± 1.5	21.0 ± 1.5	1.5	2.1	-2.8	5.8	0.491
Question 13 Recovery from fatigue (fatigue relieved – not relieved)	16.8 ± 5.2	17.6 ± 6.7	17.2 ± 6.5	20.0 ± 6.8	17.4 ± 1.3	19.8 ± 1.3	2.5	1.8	-1.2	6.1	0.181
Question 14 Feeling refreshed upon awakening (refreshed – not refreshed)	14.3 ± 4.1	14.8 ± 5.8	16.5 ± 6.2	19.5 ± 4.7	16.6 ± 1.0	19.5 ± 1.0	2.8	1.4	0.0	5.7	0.054
Question 15 Sleep duration (sufficient – insufficient)	14.8 ± 5.4	14.6 ± 3.6	18.9 ± 4.1	19.1 ± 5.0	18.9 ± 0.8	19.1 ± 0.8	0.2	1.2	-2.2	2.6	0.839
Question 16 Sleep continuity (present – absent)	14.8 ± 4.6	13.4 ± 3.6	16.2 ± 5.9	19.8 ± 5.4	16.1 ± 1.1	19.9 ± 1.1	3.8	1.6	0.6	7.1	0.023

Baseline and week-4 values are presented as mean ± SD. Adjusted week-4 values are estimated marginal means (EMMs) ± standard error (SE) from ANCOVA with baseline as a covariate and group (Placebo, SNK) as a fixed factor. Δ denotes the between-group difference (SNK − Placebo) in adjusted means with its 95% confidence interval (CI); SE for Δ is also reported. All p-values are two-sided with $\alpha = 0.05$. Analyses were performed in the PPS. Analysis-set sizes: Placebo, $n = 25$; SNK, $n = 25$.

Abbreviations: SNK, heat-killed *Lactiplantibacillus plantarum* SNK12; OSA-MA, Oguri–Shirakawa–Azumi Sleep Inventory MA.

Table S2. Safety outcomes at week 4: urinalysis and blood chemistry.

			between-group comparison						
Parameter	Placebo (n, %)	SNK (n, %)	OR	95% CI-	95% CI+	Δ (%)	95% CI-	95% CI+	p-value
Urine protein (qualitative)	0 (0.0)	0 (0.0)	NA	NA	NA	0	NA	NA	NA

Urine glucose (qualitative)	0 (0.0)	0 (0.0)	NA	NA	NA	0	NA	NA	NA
Urine pH	0 (0.0)	0 (0.0)	NA	NA	NA	0	NA	NA	NA
Urine occult blood (qualitative)	0 (0.0)	3 (10.7)	NA	NA	NA	10.7	-1.3	22.7	0.080
White blood cell count (WBC)	2 (7.4)	0 (0.0)	NA	NA	NA	-7.4	-17.3	2.5	0.142
Red blood cell count (RBC)	2 (7.4)	2 (7.1)	1.0	0.1	7.4	-0.3	-14.0	13.5	0.970
Hemoglobin (Hb)	2 (7.4)	2 (7.1)	1.0	0.1	7.4	-0.3	-14.0	13.5	0.970
Hematocrit (Hct)	2 (7.4)	1 (3.6)	0.5	0.0	5.4	-3.8	-15.8	8.2	0.531
Platelet count (PLT)	0 (0.0)	1 (3.6)	NA	NA	NA	3.6	-3.5	10.6	0.322
Aspartate aminotransferase (AST; GOT)	0 (0.0)	0 (0.0)	NA	NA	NA	0	NA	NA	NA
Alanine aminotransferase (ALT; GPT)	0 (0.0)	1 (3.7)	NA	NA	NA	3.6	-3.5	10.6	0.322
Gamma-glutamyl transferase (GGT; γ-GTP)	0 (0.0)	0 (0.0)	NA	NA	NA	0	NA	NA	NA
Total bilirubin (T-Bil)	0 (0.0)	1 (3.6)	NA	NA	NA	3.6	-3.5	10.6	0.322
Total protein (TP)	3 (11.1)	2 (7.1)	0.6	0.1	4.0	-4.0	-19.2	11.2	0.609
Urea nitrogen (UN) †	2 (7.4)	1 (3.6)	0.5	0.0	5.4	-3.8	-15.8	8.2	0.531
Creatinine (CRE)	2 (7.4)	2 (7.1)	1.0	0.1	7.4	-0.3	-14.0	13.5	0.970
Uric acid (UA)	0 (0.0)	1 (3.6)	NA	NA	NA	3.6	-3.5	10.6	0.322
Sodium (Na)	0 (0.0)	0 (0.0)	NA	NA	NA	0	NA	NA	NA
Potassium (K)	3 (11.1)	0 (0.0)	NA	NA	NA	-11.1	-23.1	0.89	0.070
Chloride (Cl)	0 (0.0)	0 (0.0)	NA	NA	NA	0	NA	NA	NA
Serum amylase (AMY)	0 (0.0)	2 (7.1)	NA	NA	NA	7.1	-2.8	17.0	0.157
Total cholesterol (T-Cho)	4 (14.8)	3 (10.7)	0.7	0.1	3.4	-4.1	-21.7	13.5	0.648
HDL cholesterol (HDL-Cho)	1 (3.7)	2 (7.1)	2.0	0.2	23.4	3.4	-8.6	15.4	0.574
LDL cholesterol (LDL-Cho)	5 (18.5)	3 (10.7)	0.5	0.1	2.5	-7.8	-26.4	10.8	0.412
Triglycerides (TG)	1 (3.7)	0 (0.0)	NA	NA	NA	-3.7	-10.8	3.4	0.304

Glucose (GLU)	1 (3.7)	1 (3.6)	1.0	0.1	16.2	-0.1	-10.0	9.8	0.979
Hemoglobin A1c (HbA1c; NGSP)	0 (0.0)	0 (0.0)	NA	NA	NA	0	NA	NA	NA

Values are shown as n and a percentage. The group indicates randomized allocation (Placebo, SNK). Analysis-set sizes: Placebo, $n = 27$; SNK, $n = 28$. Between-group comparisons of proportions used Fisher's exact test, or the χ^2 test when all expected cell counts were ≥ 5. Δ (percentage points) denotes the difference in proportions (SNK − Placebo). "95% CI−/95% CI+" indicates the lower and upper limits of the 95% CI. NA, not applicable (e.g., zero cells). All p-values are two-sided with $\alpha = 0.05$.

Abbreviations: SNK, heat-killed *Lactiplantibacillus plantarum* SNK12; WBC, white blood cells; RBC, red blood cells; Hb, hemoglobin; Ht, hematocrit; PLT, platelets; ALT, alanine aminotransferase; AST, aspartate aminotransferase; γ-GTP, gamma-glutamyl transpeptidase; ALP, alkaline phosphatase; T-Bil, total bilirubin; T-Cho, total cholesterol; HDL-Cho, high-density lipoprotein cholesterol; LDL-Cho, low-density lipoprotein cholesterol; TG, triglycerides; Cre, creatinine; UA, uric acid; GLU, glucose; AMY, amylase; Na, sodium; K, potassium; Cl, chloride.

† Urea nitrogen (UN) is also referred to as blood urea nitrogen (BUN) in clinical chemistry.

Table S3. Plasma IL-6 concentration at baseline and week 4, and adjusted week-4 means.

Parameter	Timepoint	Placebo (mean ± SD)	SNK (mean ± SD)	Adjusted mean (estimated marginal means (EMM) ± standard error (SE))	between-group comparison Δ (SNK − Placebo)	SE	95% CI−	95% CI+	p-value
IL-6 (pg/mL)	Pre-intervention	14.36 ± 50.46	10.02 ± 21.14	-	-4.34	10.94	-26.63	17.94	0.69
	Post-intervention	49.14 ± 181.23	8.80 ± 17.97	47.53 ± 25.40	-37.12	35.94	-109.43	35.19	0.31

Data are presented as mean ± SD at pre- and post-intervention. Adjusted post-intervention values are estimated marginal means (EMM) ± standard error (SE) from ANCOVA with baseline as a covariate and group (Placebo, SNK) as a fixed factor. Δ denotes the between-group difference (SNK − Placebo) in adjusted means with its 95% CI. All p-values are two-sided with $\alpha = 0.05$. Analyses were performed in the PPS. Analysis-set sizes: Placebo, $n = 25$; SNK, $n = 25$.

Abbreviations: SNK, heat-killed *Lactiplantibacillus plantarum* SNK12.
